# Dr. Edmund Henry Pettifer

**Published:** 1911-09-30

**Authors:** 


### OBITUARY.

The death is announced of Dr. Edmund Henry Pettifer in his eighty-fourth year. Dr. Pettifer was formerly in practice at Newington Green, London, and qualified in 1861, after pursuing his studies at St. Bartholomew's Hospital. He was a Fellow of the Hunterian Society. His death took place at Weymouth.

